# Regional difference in semen quality of young men: a review on the implication of environmental and lifestyle factors during fetal life and adulthood

**DOI:** 10.1186/s12610-020-00114-4

**Published:** 2020-10-15

**Authors:** Rita Rahban, Serge Nef

**Affiliations:** Swiss Centre for Applied Human Toxicology (SCAHT), Switzerland and Department of Genetic Medicine and Development, Faculty of Medicine, University of Geneva, Rue Michel-Servet 1, 1206 Geneva, Switzerland

**Keywords:** Semen quality, Sperm count, Regional differences, Lifestyle factors, Environmental factors, Young men, Fetal life, Adulthood, Qualité du sperme, Nombre de spermatozoïdes, Différences régionales, Facteurs liés au mode de vie, Facteurs environnementaux, Jeunes hommes, Age fœtal, Age adulte

## Abstract

The prevalence of low semen quality and the incidence of testicular cancer have been steadily increasing over the past decades in different parts of the World. Although these conditions may have a genetic or epigenetic origin, there is growing evidence that multiple environmental and lifestyle factors can act alone or in combination to induce adverse effects. Exposure to these factors may occur as early as during fetal life, via the mother, and directly throughout adulthood after full spermatogenic capacity is reached. This review aims at providing an overview of past and current trends in semen quality and its relevance to fertility as well as a barometer of men’s general health. The focus will be on recent epidemiological studies of young men from the general population highlighting geographic variations in Europe. The impact of some lifestyle and environmental factors will be discussed with their role in both fetal life and adulthood. These factors include smoking, alcohol consumption, psychological stress, exposure to electromagnetic radiation, and Endocrine Disrupting Chemicals (EDCs). Finally, the challenges in investigating the influence of environmental factors on semen quality in a fast changing world are presented.

## Résumé

La prévalence de la mauvaise qualité du sperme et l’incidence du cancer testiculaire n’ont cessé d’augmenter au cours des dernières décennies dans différentes régions du monde. Bien que ces pathologies puissent avoir une origine génétique ou épigénétique, il semble de plus en plus évident que de multiples facteurs environnementaux ou liés au mode de vie peuvent agir seuls ou en combinaison pour induire des effets délétères. L’exposition à ces facteurs peut se produire dès la vie fœtale, par l’intermédiaire de la mère, ou directement à l’âge adulte. Cette revue vise à fournir un aperçu des tendances passées et actuelles en matière de santé masculine, en se concentrant principalement sur des études épidémiologiques portant sur des jeunes hommes de la population générale et mettant en évidence les variations géographiques en Europe. L’impact de certains facteurs liés au mode de vie et à l’environnement sera examiné, ainsi que leur rôle dans la vie fœtale et à l’âge adulte. Ces facteurs comprennent le tabagisme, la consommation d’alcool, le stress psychologique, l’exposition aux rayonnements électromagnétiques et les perturbateurs endocriniens (PDE). Finalement, nous présentrons les défis auquels sont confrontés les chercheurs explorant l’impact des facteurs environnementaux sur la qualité du sperme dans un monde en rapide mutation.

## Past and present trends in semen quality

Humans have by far the lowest reproductive traits and remarkably poor fertility compared to other mammals in the animal kingdom. Although some sperm parameters are similar compared to other species, human males have markedly smaller relative testis size and the lowest rate of daily sperm production per gram testis [1]. Moreover, sperm production, or spermatogenesis, is admitted to be relatively inefficient in men since most of the produced sperms are classified as morphologically abnormal (96% according to most recent reference values of the World Health Organization –**WHO**- [2]). In domestic animals such as bulls and rams as well as in rodents, only 10% are usually classified as abnormal [3]. This suggests that spermatogenesis in men is particularly vulnerable to external factors and that humans are more likely to be at greater risk from toxic agents [1, 3–6]. The poor semen quality is manifested by the relatively high number of infertility cases affecting approximatively 15% of couples worldwide [2, 7, 8]. Concerns about a decline in semen quality in general and sperm count, in particular, are rising and had already begun as early as 1980 [9]. A secular decline over 45 years was already suspected to have occurred in men that are unselected for their fertility status [10]. This trend was further evaluated in the following years in different parts of the world and on a population of infertile men but remained controversial as will be discussed in the section below [9, 11].

### The decrease in sperm counts: a brief historical overview

In an attempt to better describe the decrease in sperm count, Carlsen et al. conducted a meta-analysis of 61 studies published between 1938 and 1990 including a total of 14,947 men with no previous history of infertility [12]. Carlsen and colleagues were one of the first to show strong evidence of a significant decline in mean sperm concentration over 50 years with values ranging from 113 Mio/mL in 1940 to 66 Mio/mL in 1990, corresponding to approximatively 1% decrease per year. These surprising values initiated a long and lively debate in the scientific community on the validity of these trends [13]. One of the major issues that has been strongly criticized is the heterogeneity of the men in terms of age, fertility and socio-economic status [14]. Important confounding factors were also thought not to be adjusted for in the Carlsen study such as the period of sexual abstinence, geographic variations and methodological differences in semen analysis [15, 16]. In fact, it has been suggested that the observed trend may reflect geographical variations rather than a decrease in sperm concentration, particularly since most of the studies before 1970 that were included in the Carlsen’s analysis were conducted in the United States [17]. Nearly a decade after its publication, Carlsen’s meta-analysis remained highly controversial and a decrease in sperm count was still heavily debated.

In order to clarify the situation regarding declining sperm count trends, Swan et al. reanalyzed the Carlsen’s data and adjusted for the period of abstinence, age, proven fertility, and methodology [18]. The authors confirmed a significant decline in median sperm count of 1.5% per year in the United States between 1938 and 1988. In addition, a decrease of about 3% per year was observed in Europe and Australia. These values were slightly higher than the average decrease of 1% per year reported by Carlsen. Swan and colleagues however, were unable to confirm a decline in non-Western countries due to very limited data availability [18]. A few years later, Swan et al. published an updated meta-analysis by including more studies using similar analysis methods and adjusting strategies [19]. A total of 47 publications in English language published between 1934 and 1996 were added to those previously analyzed. The average decline was confirmed and was virtually unchanged from that previously reported by Carlsen et al. The slope of the decrease in the U.S. was less than the 3% previously reported by Swan in 1997 and the decline in Europe was closer to that originally reported by Carlsen et al. [19]. Swan concluded by predicting that the debate on the declining sperm count trends will continue and that further statistical analysis of historical data is unlikely to resolve the controversy as critics will continue to question the reliability of data collected in a different scientific era. Indeed, the controversy over declining sperm count has continued and numerous reports questioning these trends have been published [14, 20–23]. These reports acknowledged the difficulty in controlling some confounding factors in the highly variable nature of semen analysis, collection criteria, comparability of the population from different time periods, quality assessment of laboratory methods to count sperm and, to add another layer of complexity, the potential geographic variations in semen quality [14]. Their main conclusion was that there is insufficient evidence to confirm a global decline in sperm counts.

It is only until very recently that important pieces of the puzzle have been added to the mystery of the declining trend. A meta-analysis published by Levine et al. in 2017 included data from 185 studies of 42,935 men who provided semen samples between 1973 and 2011 [24]. The main outcomes revealed a significant 52.4% decrease in sperm concentration among ‘Unselected Western’ men with the mean sperm concentration declining by 1.4% per year. A decline in sperm concentration was also observed in ‘Fertile Western’ men, while no significant trends were seen among ‘Unselected Other’ and ‘Fertile Other’ men [24]. Interestingly, there was no sign of ‘leveling off’ in the observed decline during that same period.

### Why does ‘sperm count’ count?

Spermatozoa are produced continuously in the testes by a complex cellular process called spermatogenesis involving a mitotic, meiotic and spermiogenesis phase. This process begins at puberty and continues throughout a man’s life, with each spermatogenesis cycle lasting approximately 74 days [25–27]. Sperm count, among other sperm parameters, is clearly an important indication of a men’s potential fertility status. But is that everything sperm count can predict? Multiple epidemiological studies performed on large cohorts of men revealed that sperm count is a barometer of overall health. In addition, a men’s semen quality can be predictive of other male reproductive disorders as will be discussed below.

#### Prediction of male fertility

Two main factors determine a man’s sperm count at any given time: the total number of Sertoli cells present in his testes and the time since the last ejaculation [5]. While the abstinence is variable, the number of Sertoli cells is determined during testis development prior to puberty [28–30]. Assessment of sperm count as well as sperm motility and morphology, is the first step and the mainstay in identifying male factor infertility. Semen quality analysis has been standardized thanks to the efforts of the WHO to produce and publish practical manuals since the 1970s with the 5th and latest edition being published in 2010 [31]. The purpose of this manual is to improve the standards of semen analysis and ensure that scientists use standardized methods. This has enabled the comparison of semen quality worldwide on large and diverse data sets, paving the way for numerous epidemiological studies. However, semen quality reference values dictated by the WHO cannot predict the fertility of a man. This is because, besides the contribution of the partner’s fertility, there are many factors that contribute to the ability of spermatozoa to fertilize an egg. The WHO initially adopted a sperm concentration of < 20 Mio/mL and 40 Mio sperm in the ejaculate as thresholds below which men were considered subfertile [32] based on studies conducted in the 1950s by MacLeod and colleagues [33, 34]. Little was known about the relationship between semen parameters and time to pregnancy (**TTP**) until epidemiological studies assessing the likelihood of a woman becoming pregnant based on her partner’s semen parameters were undertaken. It was not until 2001 that Guzick and colleagues questioned the clinical significance of these values and analyzed semen samples from male partners in 765 infertile couples and 693 fertile couples [35]. These studies suggested that the lower threshold of sperm concentration for subfertility was 13.5 Mio/mL and the fertile ranges were a concentration of more than 48 Mio /mL [35]. In another observational study, Slama et al. analyzed data of partners of pregnant women from four European countries and showed that the probability of becoming pregnant increased with increasing sperm concentration up to 55 Mio/mL [36]. A linear relationship between the increasing sperm concentration and the percentage chance of pregnancy was observed. In other words, as sperm concentration rises from zero to around an average of 40 Mio/mL, the partner’s chances of becoming pregnant progressively increase. Similarly, it has been shown that the proportion of morphologically normal sperms is strongly related to the likelihood of pregnancy regardless of sperm concentration [35–39]. Overall, the literature strongly suggests that the assessment of semen quality, especially sperm concentration, morphology, and total sperm count are highly predictive of men’s fecundity and fertility. The high economic and societal burden of male infertility, which continues to grow, emphasizes the importance of semen quality assessment [40].

#### Indication of the general health status

It has been suggested that semen quality is a fundamental biomarker of overall male health [41]. Semen analysis data on 43,277 men who attended fertility clinics between 1963 and 2001 were associated with available information on the causes of their illness, cancer, death and the number of children they had. Among men without azoospermia, mortality decreased as the sperm concentration increased to a threshold of 40 Mio/mL. It has been shown that the percentage of motile and morphologically normal spermatozoa as well as semen volume increased with decreasing mortality [41]. In another study on 11,935 men evaluated for infertility from 1989 to 2011 in the United States, it was found that men with abnormal semen parameters had a higher risk of death, suggesting a possible common etiology between infertility and mortality [42]. Men diagnosed with infertility were also shown to have a higher risk of developing diabetes, ischemic heart disease, and suffer from drugs and alcohol abuse [43]. In a more recent study evaluating the relationship between semen quality and morbidity, a clear association between sperm concentration below 15 Mio/mL and all-cause hospitalization and cardiovascular diseases was found compared with men with a concentration above 40 Mio/mL. Semen quality was therefore associated with long-term morbidity and a significantly higher risk of hospitalization [44]. A potential explanation of the link between male factor infertility and future adverse health outcomes could be hormonal. Infertle men with low sperm count have more often lower total circulating testeosterone levels than fertile men [45]. Since hypogonadism is considered to be a risk factor for cardiovascular diseases and mortality, low testerone levels can link infertility to mortality. Even though sperm count can be a marker of dimished fitness, it might also occur as a consequence of current health conditions [43].

#### Association with male reproductive disorders

The decline in sperm count that has been reported in various geographic regions is not the only observation in the field of male reproductive health. Concomitantly, an increase in genital malformations (such as cryptorchidism and hypospadias) and testicular cancer has also been reported. Described for the first time in 2001, the Testicular Dysgenesis Syndrome (**TDS**) was one of the first hypotheses attempting to explain the various negative trends observed in male reproductive health. Specifically, this hypothesis suggests that the common cause is exposure to environmental and lifestyle factors during fetal life [46]. It proposes that disturbed testicular development in utero during the specific window called the masculinization programming window (**MPW** - around week 8–14 of gestation in humans) may result in the occurrence of one or a combination of reproductive disorders such as cryptorchidism, hypospadias, low sperm count and testicular germ cell cancer [46]. This hypothesis was built based on multiple clinical, epidemiological, and experimental observations and was corroborated over the last two decades by new evidence from toxicological, biological, and genetic data. A fetal origin is clear with regard to hypospadias and congenital cryptorchidism. However, since both semen quality and testicular cancer are only manifested in adulthood, the link with a fetal origin was more challenged [47]. The observation of considerable geographic variation in sperm count and testicular cancer suggest the involvement of different environmental exposures [48].

## Geographical variations in semen quality

One of the first indications of geographic variations in semen quality emerged following the previously described meta-analysis of Carlsen in the 1990’s [12]. The study has prompted many scientists around the world to asses temporal trends of semen quality in their countries [17]. Most of these studies were conducted on infertile men, semen donors, or male partners of infertile women. While the decrease in sperm count remained controversial, the geographic differences were not. These studies all agreed that the median sperm count varies greatly among comparable populations in different countries. However, geographic variations could not be assessed due to different recruitment strategies, semen analysis methods, and population selection. Evidence of geographic variations was mainly provided by cross-sectional studies using standardized methods for semen analysis and similar population selection strategies. In the following section, the semen quality of young men without prior knowledge of their fertility status, recruited in similar fashion and considered to be representative of the general population will be examined and discussed. At the age of 20, men have reached full spermatogenic capacity and sperm numbers remain fairly constant during their third decade [49]. Cross-sectional data on young men do, therefore, represent their adult sperm production. A comprehensive list including comparative studies on fertile men and sperm bank donors in the twenty-first century has been reviewed [50].

### Young men from the general population

**Fig. 1:**
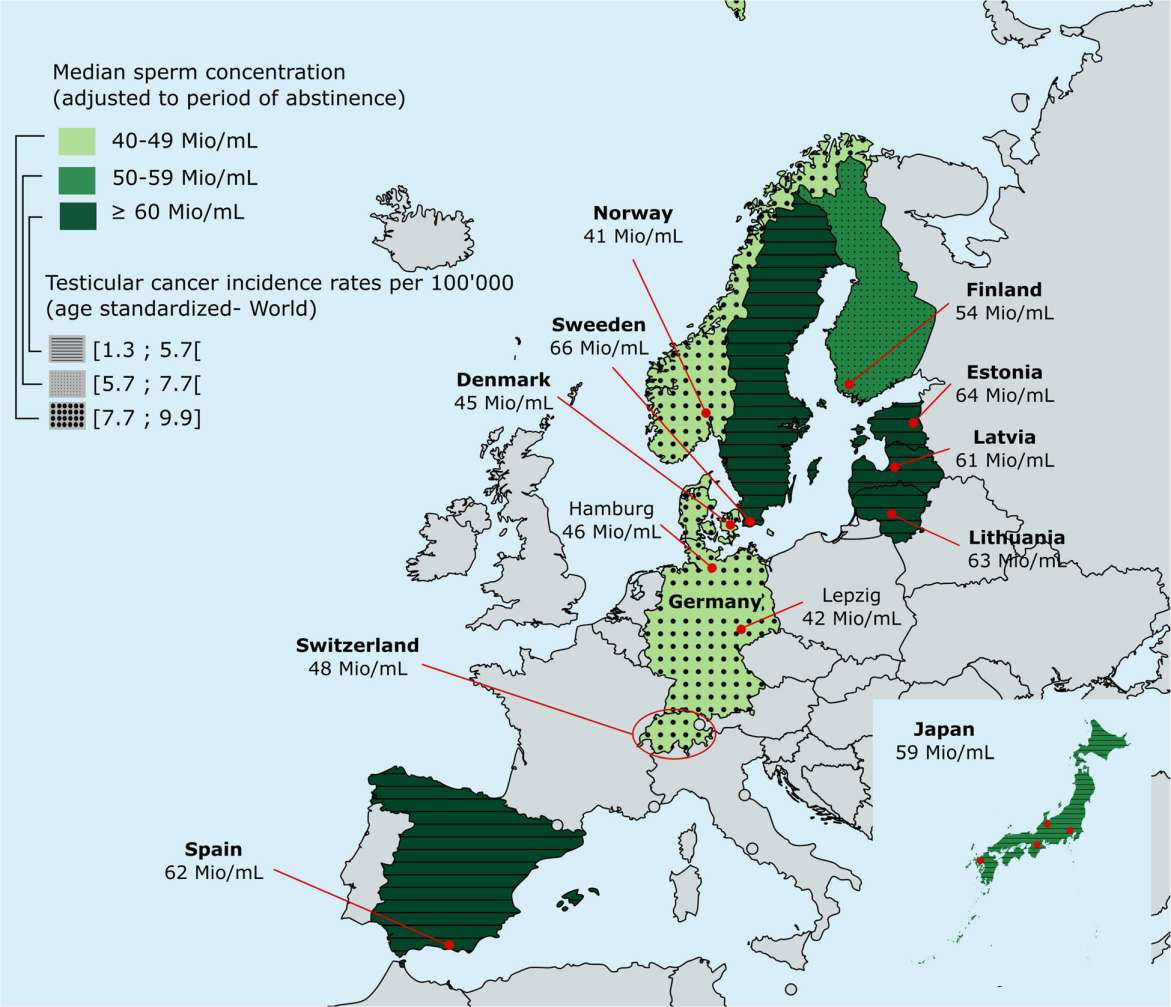
Regional differences in semen quality and in testicular cancer rates. Median sperm concentrations of young men (adjusted for a period of sexual abstinence of 96 h) are indicated in a color gradient, with the darkest green corresponding to the group of countries with the highest sperm concentration. Testicular cancer incidence rates (age-standardized World per 100′000) in Europe and Japan are also shown with different patterns. Red dots indicate the cities where the studies were performed and the circle around Switzerland refers to the whole country. The map was adapted from [50] and the sources of data on sperm concentration were extracted from [51–55, 56] and as personal communications from J. Axelsson and J. Erenpreiss. Cancer data were obtained from [57]. A correlation between sperm concentration and the incidence of testicular cancer in Europe was observed using a Spearman Rank Order Correlation (r = 0.74, *P* = 0.01). Mio/mL: Million/milliliter

The three folds higher incidence of testicular cancer in Denmark and Norway compared to Estonia and Finland was one of the main reasons that prompted scientists to evaluate semen quality among young men in these four different geographic regions [51]. The aim was to evaluate whether low semen quality is correlated with high rates of testicular cancer as the TDS hypothesis suggests. This study was one of the first to be coordinated and conducted under the same protocols and with appropriate quality control and quality assessment procedures among four laboratories [51]. The men participating in the study were considered to be representative of the general population in the four countries since they were recruited during military conscription and were not selected according to their fertility status. A total of 968 young men were recruited and results revealed that median sperm concentrations were significantly higher among Finnish and Estonian men (54 and 57 Mio/mL, respectively) compared to Danish and Norwegian men (41 Mio/mL) after adjustment to the Danish laboratory level and period of sexual abstinence (Fig. 1) [51]. Similarly, total sperm count was classified from the highest to the lowest values as follows: Finish (185 Mio), Estonian (174 Mio), Danish (144 Mio) and Norwegian (133 Mio). It was therefore concluded that there is an East-West gradient in semen quality in the Nordic-Baltic area. Two other studies followed this coordinated evaluation, one comparing Swedish and Danish men [58] and the other comparing Estonian and Lithuanian men [59]. Young Swedish men were found to have a significantly higher sperm concentration than Danish men with a median of 55 Mio/mL and a mean difference of 13.4 Mio/mL (Fig. 1, Table) [58, 60]. Although the studies had identical recruitment strategies, no reference laboratory was used in the Swedish study and potential inter-laboratory differences were not taken into account when calculating differences in sperm concentration. In the comparative study between men from two Baltic countries, median sperm concentration (adjusted for inter-laboratory differences and abstinence period) was higher in Estonian men compared with Lithuanians (67 and 55 Mio/mL, respectively) (Fig. 1, Table) [59]. The evaluation of sperm concentration among Latvian military conscripts (with a median of 63 Mio/mL) was shown to be very similar to one reported in Estonia (median of 67 Mio/mL) among men of similar age, but slightly higher than in Sweden and significantly higher than in Denmark [61, 62]. However, it is noteworthy to mention that these results were obtained on a small number of men (133) and have not been adjusted to other reference laboratory levels such as those in Danemark. In a more recent study in southern Sweden, 295 young men were recruited between the years 2008 and 2010 in order to compare them with the previous cohort of 216 men analyzed in 2002 [58, 63]. The results revealed that sperm concentration did not deteriorate over almost a decade with a median sperm concentration of 56 Mio/mL [63]. These studies conducted in Scandinavian countries and the Baltic area raised concerns about semen quality of young men in a large variety of countries and numerous cohort studies on young men followed in the rest of Europe as well as in Japan. German young men from Leipzig and Hamburg had a median sperm concentration similar to men in Denmark and Norway with an adjusted median sperm concentration of 42–46 Mio/mL [52]. However, the adjusted sperm concentration was found to be higher in southern Spain and in four Japanese cities (Kawasaki, Osaka, Kanazawa, and Nagasaki) with values ranging from 62 to 59 Mio/mL, respectively (Fig. 1, Table) [53, 54]. Semen quality in the Faroe Islands in the North Atlantic, halfway between Norway and Iceland, was also evaluated as these islands are highly exposed to persistent organic pollutants from traditional marine food and low values were suspected. Indeed, crude median sperm concentrations of Faroese men was lower than that of Danish men (40 vs 48 Mio/mL) [64]. Across the Atlantic, a study on young men in New York revealed that median sperm concentration was 52 Mio/mL, higher than Danish and Finnish men and lower than Japanese men [65]. On the other side of the globe, 423 young men participated in an Australian birth cohort called Raine aimed at evaluating testicular functions [66]. The median sperm concentration of men was 45 Mio/mL and was associated with the occurrence of varicocele, cryptorchidism and a significant reduction in testicular volume.

Recent studies in Scandinavian countries revealed that the difference between Finland and Denmark is narrowing down, as sperm concentrations in Finland are decreasing and those in Denmark are increasing [67]. When excluding men with previous or current andrological disorders, these values did not seem to change and the Danish increase remained statistically significant (*p* = 0.02 for sperm concentration in 1996–2000 vs 2006–2010). These values have not changed in almost a decade despite a reduction in maternal smoking that was often associated with decreased sperm counts [55]. Another recent update on semen quality among young Finnish men compared to Danish men revealed that the adjusted median sperm concentration in Finland remains slightly higher (49 vs 47 Mio/mL, respectively) [68]. In the Baltic area, median sperm concentration values for Estonians, Latvians, and Lithuanians were found to be very similar (63, 55, and 63 Mio/mL, respectively) [69].

Recently, the first study evaluating the semen quality of young men on a national level - and not only on a regional level as previously performed - was published in Switzerland [56]. A total of 2′523 volunteers representative of the male population in the country was evaluated. The median sperm concentration (48 Mio/mL) was comparable to the values previously published in Germany. An evaluation of geographical factors, urbanization rates or linguistic regions as a way to differentiate lifestyle habits revealed no major differences in semen quality. Testicular cancer incidence rates in the general Swiss population were also shown to have increased significantly in the past 30 years. A correlation with the low median sperm concentration was found to be significant (Fig. 1).

## Lifestyle and environmental factors during fetal life and in adulthood

**Fig. 2:**
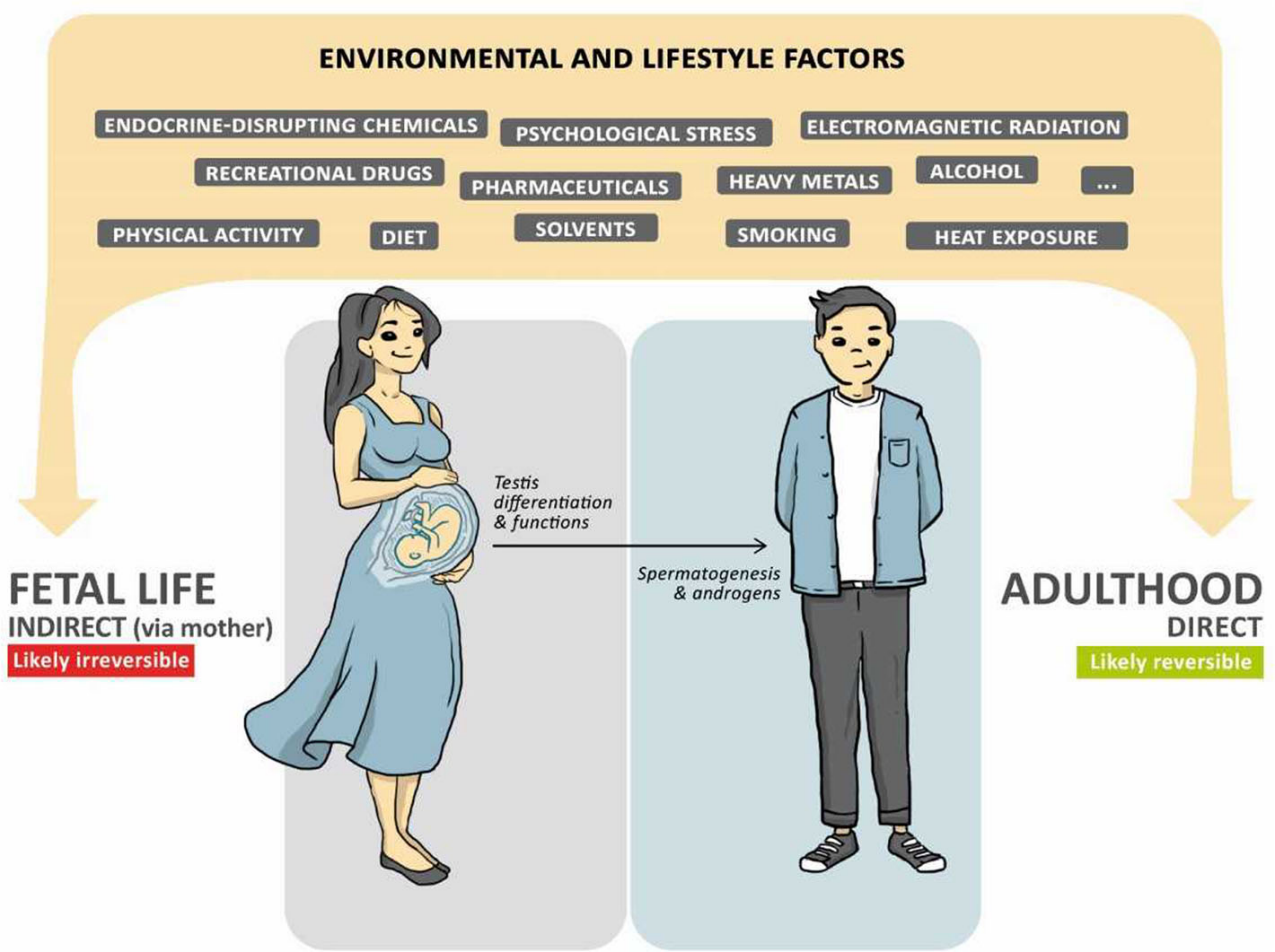
Influence of environmental and lifestyle factors throughout a man’s life. Numerous environmental or lifestyle factors can affect testicular development and function during both fetal life and adulthood. Exposure to these factors via the mother during pregnancy can affect the differentiation and/or endocrine functions of the fetal testis resulting in a reduced synthesis of androgens by Leydig cells and/or reduction in the final number of Sertoli cells. This can lead to an irreversible reduction in sperm count and sperm fertilizing capacities in adulthood. In adult men, the effects of environmental or lifestyle factors can affect spermatogenesis and/or the production and action of androgens. However, the impact is likely to be reversible because a new cycle of spermatogenesis is taking place approximatively every 74 days

Numerous environmental factors and lifestyle habits have been described to affect a men’s reproductive health as early as during fetal life and throughout adulthood (Fig. 2). Evaluation of some of their impact on semen quality received considerable attention after the strong decline in sperm count was described [12]. Some of the most studied and relevant factors will be discussed below such as smoking, alcohol consumption, stress, and exposure to electromagnetic radiation. Evidence of exposure during fetal life will be provided when available but for most of the factors, exposure was evaluated in adulthood.

### Smoking

The tobacco epidemic is one of the biggest public health threats in the world with more than 8 million people dying each year due to tobacco-related illnesses such as cancers, cardiovascular diseases, diabetes and stroke [70]. Besides its disastrous effects on overall health, tobacco consumption during adulthood has been recognized as a risk factor of male infertility [71]. A systematic review evaluating the relationship between lifestyle factors and semen quality showed a significant association between smoking and semen volume, sperm concentration, total sperm count, sperm motility as well as sperm morphology [72, 73]. A systematic review followed by a meta-analysis also showed a clear association between reduced sperm concentration, motility and morphology, and cigarette smoking [74]. In another type of study, sperm aneuploidy was evaluated in relation to cigarette smoking. A statistically significant increase in sperm disomy among smokers was observed compared with non-smokers [75]. In an evaluation of sperm DNA, fertile smokers were significantly associated with higher fragmentation and higher seminal reactive oxygen species (ROS) levels [76].

Prenatal exposure to maternal smoking has also been repeatedly shown to be associated with reduced semen quality [71, 77–80] and this has been recently shown to be translated by a reduced men’s fertility [81]. A study on 347 Danish young men revealed an inverse association between maternal smoking during pregnancy and total sperm count, sperm concentration and semen volume [79]. A cross-sectional study on 1′770 young men from the general population in five European countries (Denmark, Norway, Finland, Lithuania, and Estonia) showed a significant association between in utero exposure to maternal smoking and reduced semen quality as well as testicular size in adulthood [82]. A more recent study on 537 Argentinian men also shows that maternal tobacco consumption during pregnancy was associated with a significantly higher risk of reduced sperm count and elevated total testosterone levels [83]. Interestingly, men prenatally exposed to smoking are more likely to be smokers themselves [79]. In another study involving both parents, paternal smoking was associated with 46% lower total sperm count in maternally unexposed men and both paternal and maternal smoking was associated with a lower sperm concentration [84]. Tobacco contains numerous hazardous substances and the mechanism(s) of action mediating adverse effects is difficult to elucidate. Oxidative stress, DNA damage, cell apoptosis and a direct effect on the regulation of spermatogenesis have all been suggested as potential mechanisms [3, 85, 86].

### Alcohol

Chronic and acute alcohol abuse is involved in the pathogenesis of many diseases, including liver and cardiovascular diseases, cancers as well as neuropsychiatric disorders to name a few. Alcohol consumption has been shown to be much higher among men compared to women [87]. However, relatively few studies have examined the correlation between alcohol consumption and male reproductive functions [88, 89]. Moreover, most of these studies have been conducted in selected populations of infertile men or have a small sample size, with conflicting results [90]. In the same large meta-analysis that evaluated the effect of smoking on adult men, the authors also examined the association with alcohol consumption and found that it is negatively associated with semen volume but not with other measures of semen quality [72]. However, in this analysis, only four studies have been included. A large cross-sectional study was initiated a few years later aiming at evaluating more closely the link between alcohol consumption and semen quality [90]. This study was performed on 8344 healthy young men from Europe and the United States who all completed a questionnaire on health and lifestyle including their intake of beer, wine, and liquor during the week prior to their visit. Moderate alcohol consumption was not adversely associated with semen quality but was associated with higher serum testosterone levels [90]. However, another cross-sectional study by the same author that was carried out on 1221 young Danish men found that the habitual consumption of alcohol was associated with reduced sperm concentration, decreased total sperm count, and reduced normal sperm morphology [88]. This association was more pronounced for men with a typical intake of more than 25 units of alcohol per week, one unit being equivalent to 12 g of ethanol [88]. A more recent systematic review followed by a meta-analysis involving 15 cross-sectional studies with 16,395 enrolled men showed that alcohol intake has a detrimental effect on semen volume and normal sperm morphology but not on sperm concentration nor sperm motility [91]. The difference was more pronounced when comparing occasional versus daily consumers, rather than never versus occasional, suggesting that a moderate consumption does not adversely affect semen parameters [91]. This was subsequently confirmed by the same author in a cross-sectional analysis on men from an Italian fertility clinic [92]. In a prospective autopsy study designed to assess differences in testicular histology of heavy drinkers compared to moderate or non-drinkers, spermatogenic arrest and ‘Sertoli-cell only’ (**SCO**) syndrome was shown to be present in 50 and 10% of heavy drinkers, respectively [93]. A dose-dependent association between spermatogenic arrest and alcohol consumption was later confirmed with a significantly increased risk in men who consumed an average of 80 g per day [94]. The spermatogenic damage caused by alcohol abuse, however, has been shown to be reversible. Case reports, as well as animal studies, showed that spontaneous recovery of spermatogenesis could occur after 10–12 weeks of alcohol withdrawal, equivalent to one cycle of spermatogenesis [95, 96]. Besides its adverse effect on spermatogenesis, alcohol has also been shown to decrease testosterone blood concentration by acting both on testicular and central (hypothalamic and pituitary) levels [97]. Indeed, alcohol was shown to exert an inhibitory action on the enzymes that catalyze the conversion of pregnenolone to progesterone and androstenedione to testosterone (3 β-hydroxysteroid dehydrogenase and 17-ketosteroid reductase, respectively) [98]. Alcohol was also shown to be associated with the induction of sperm aneuploidy [75].

The relationship between prenatal alcohol exposure and adult semen quality has also been evaluated but studies are very limited and results are conflicting [89]. In a follow-up study of a cohort of Danish pregnant women, sperm concentration decreased with increasing prenatal alcohol exposure [99]. No associations were found for sperm motility, sperm morphology or any of the reproductive hormones including testosterone. The proposed mechanism was suggested to involve persistent adverse effects on Sertoli cells.

### Stress

The impact of psychological stress on semen quality is of central importance but is nevertheless challenging to asses. During adulthood, the impact is thought to alter spermatogenesis [100, 101]. However, exposure during fetal life can be more detrimental since it might impact the androgen activity and testicular development [102, 103]. The same meta-analysis evaluating the effects of smoking and alcohol on semen quality in 2011 also evaluated the effect of different forms of psychological stress [72]. The study found that stress might be associated with reduced sperm concentration, progressive sperm motility and abnormal sperm morphology. A similar result was found in another large cross-sectional study of young Danish men from the general population. The studyrevealed a negative association between self-reported stress and semen volume, sperm concentration, total sperm count, and morphologically normal sperms [100]. Men with the highest stress level had 38% lower sperm concentration, 34% lower total sperm number and 15% lower semen volume compared to men with intermediate stress levels. It has been therefore suggested that stress exerts an adverse effect on semen quality by inducing apoptosis of sensitive germ cells via high levels of glucocorticoid although the mechanism of action is certainly more complex [100].

Extensive animal data suggest that maternal stress during pregnancy can have a negative impact on male reproductive functions in adult male offsprings. In particular, it can lead to reduced fertility, sexual activity, fewer ejaculation, decreased testicular weight and delayed puberty [104]. However, human evidence regarding the association between maternal stressful life events (**SLE**) and male reproductive functions are very sparse. A Danish nation-wide cohort study evaluated this association with the prenatal stress exposure being the mother’s loss of a close relative during pregnancy or in the 12 months before conception [105]. Prenatal exposure to stress was significantly associated with an elevated risk of congenital malformations and infertility. A more recent prospective study in Australia (the Raine study) found that exposure to SLE, in early but not late gestation, was associated with reduced adult male reproductive functions such as total sperm count, number of progressively motile sperms and morning serum testosterone concentration [106]. How paternal SLE affect male reproductive function is less considered. An emerging number of evidence suggests a paternal influence on the offspring’s reproductive fitness. Genetic susceptibility or epigenetic modifications are thought to be important mediators explaining interactions between a stressful environment and sperm/offspring outcomes [107].

### Mobile cell phone use

The use of mobile phones has increased considerably over the past decade and concerns are growing about the possible detrimental effects of high-frequency electromagnetic fields (**EMF**) emitted by these devices on human health. The type of EMF phones emit are low-level Radio Frequency (**RF-EMF**) (850 MHz-2.4 GHz) that can be absorbed by the human body [108]. Very few studies aimed at evaluating their effects on reproductive health have been conducted, and the majority was performed on a small sample of men. In an observational study on 361 men attending an infertility clinic, mean sperm motility, viability, and normal sperm morphology were significantly lower in men with increasing daily exposure to cell phones [108]. The authors suggested that this might contribute to male infertility. Following these studies, the direct effect of the RF-EMF was tested in vitro to evaluate the direct effect on sperm quality. Reactive oxygen species (**ROS**) levels were measured and RF-EMR were shown to induce DNA damage due to increased levels of oxidative stress which was suggested to accelerate sperm cell death and promote testicular carcinogenesis [109, 110]. In another prospective in vitro study, a total of 124 semen samples were exposed to 1 h of cell phone radiation and sperm parameters were recorded before and after exposure. A significant decrease in sperm motility, sperm linear velocity, and acrosome reaction, as well as a significant increase in sperm DNA fragmentation, were observed [111]. These observations were further confirmed by another study on 32 healthy men that had their sperm sample exposed for 5 h in vitro*.* The number of sperm with progressive motility was significantly reduced in the exposed samples and a higher percentage of sperm with DNA fragmentation was observed [112]. A statistically significant decrease was also observed in the rapidly progressive and slow progressive sperms in another study on 27 men with otherwise normal sperm parameters [113]. A systematic review and a meta-analysis were recently published including data on 10 studies and a total of 1492 samples [114]. Exposure to mobile phones was mostly associated with reduced sperm motility and viability, but the effects on concentration were more equivocal. The authors suggested, however, that further studies are needed to determine the full clinical implications of these observations [114]. The use of laptop computers connected to the internet wirelessly was similarly shown to induce a decrease in sperm motility as well as an increase in DNA fragmentation [115].

The mechanism of action by which RF-EMF is suggested to affect sperm motility involves potentially an RF-induced increase in superoxide anions concentrations due to an increased level of oxidative stress [110]. These free radicals generated by sperm mitochondria are thought to oxidize membrane phospholipids resulting in decreased vitality and impaired motility [116]. In rodents, EMFs have been shown to decrease fertilization rates and spermatogenic cell numbers as well as inducing apoptosis [117–119].

Studies aimed at evaluating the relationship between maternal mobile cell phone use during pregnancy and men’s future reproductive health are very limited. A prospective study based on the Norwegian Mother and Child Cohort evaluated both parent’s exposure to RF-EMF through mobile cell phone use and pregnancy outcomes [120]. No association was found between maternal cell phone use and congenital malformation, perinatal mortality, low birth rate or change in sex ratio. Paternal pre-conceptional cell phone use was also not associated with adverse pregnancy outcomes [120].

### Endocrine disrupting chemicals (EDCs)

According to the definition of the Endocrine Society, EDCs are “exogenous agents that interfere with the synthesis, secretion, transport, metabolism, binding action or elimination of hormones present in the body and responsible for homeostasis, reproduction and developmental processes” [121]. Other definitions of the WHO and the European guidelines exist and differ slightly [122, 123]. They include the EDCs impact on the progeny and the potential effects of a combination of EDCs that if administrated alone do not necessarily have an impact. This phenomenon is referred to as the “cocktail effect” [124–126]. The group of molecules identified as endocrine disruptors is highly heterogeneous and a non-exhaustive list includes 1) industrial solvents/lubricants (polychlorinated biphenyls – **PCBs**, polybrominated biphenyls – **PBBs**, dioxins), 2) perfluorinated compounds (**PCFs**), such as perfluorinated alkyl acids (**PFAA**) 3) phenols (bisphenol A – **BPA**), 4) plasticizers such as phthalates, 5) pesticides and fungicides such as dichlorodiphenyltrichloroethane – **DDT**) and 6) pharmaceutical agents (diethylstilbestrol – **DES**), 7) UV-filters and parabens [121]. Natural chemicals can also act as EDCs such as phytoestrogens. Some of these molecules are considered to be environmentally persistent organic pollutants (**POP**) which remain intact for long periods, are widely present in the environment and are toxic [50]. POPs mainly consists of by-products from various chemicals and combustion processes of PCBs and polychlorinated dibenzofurans (**PCDF**) as well as dioxins such as polychlorinated dibenzo-p- dioxins.

Although concerns about the negative impact of EDCs on reproductive health arose a long time ago, mainly after “Silent Spring” by Rachel Carson was published in 1962, this topic is still highly controversial in the scientific field. One of the major reasons behind this debate is that most of the evidence concerning EDCs comes from extensive wildlife and animal experimental studies [121, 124, 125, 127–129]. These studies showed that fetal exposure to EDC mixtures, at doses at which individual chemicals are ineffective, can cause profound lifelong adverse effects [130, 131]. Unfortunately, similar detailed studies in humans are few and evidence on whether EDCs contribute to human health disorders is only starting to arise. These studies are challenging mainly since they are aimed at understanding the potential consequences of an event that occurred a quarter of a century earlier. A review of epidemiological studies evaluating semen quality and exposure to EDC during fetal life and adulthood will be discussed below.

#### Prenatal exposure to EDCs

One of the main problems fueling the controversy about the effects of fetal exposure to EDCs on male reproductive health is the lack of supporting evidence, particularly with respect to their adverse effects on sperm counts [3, 5, 6, 132]. In the public eye, there is probably no doubt that exposure to EDCs during fetal life accounts for falling sperm counts but in fact there are very few scientific studies demonstrating such a link. The most famous example is related to the explosion of a trichlorophenol manufacturing plant near Seveso, Italy, in 1976 releasing up to 30 kg of 2,3,7,8-tetrachlorodibenzo-p-dioxin (**TCDD**) [133, 134]. TCDD is a highly toxic by-product of combustion processes such as incineration that is known to accumulate in the human body. A study on men from Seveso provided evidence of a permanent disruptive effect of TCDD on the human male reproductive system depending on the age of exposure. Men who were exposed to TCDD when they were below the age of 9 had a reduced sperm concentration and motility compared to men who were not exposed. However, when exposure occurred at an average age of 21, no effects were observed on the 40 years old men [133]. A few years later, the same group evaluated the relation between perinatal exposure to TCDD during pregnancy and human semen quality during adulthood [134]. They observed that exposure to relatively low levels of dioxins, in utero and via lactation, can permanently reduce sperm quality [134]. In another large-scale poisoning that occurred in central Taiwan in 1979 following ingestion of cooking oil contaminated by polychlorinated biphenyls (**PCB**s) (YuCheng accident), prenatally exposed men were shown to have increased abnormal sperm morphology, reduced sperm motility and reduced sperm capacity to penetrate a hamster oocyte [135]. The authors were unable to conclude whether this exposure will lead to a reduced fecundity and how these effects can be extrapolated to the general population. A study aimed at evaluating if maternal serum concentrations of PCBs and diphenyl-dichloro-ethylene (**DDE**) during pregnancy are associated with the son’s semen quality level suggested that measured EDCs were not significantly associated with semen quality [136]. In another study by the same author, in utero exposure to PFAAs that also belong to the persistent organic pollutants (**POPs**) family, was associated with lower adjusted sperm concentration and lower total sperm counts [137]. This suggests that levels associated with adverse effects vary between chemicals, adding another layer of complexity to the EDCs hypothesis. In a recent study, Hart et al. evaluated the relationship between prenatal maternal exposure to BPA or phthalates and semen quality of the sons at age 20–22 years in the Raine pregnancy cohort study [138]. The authors found that after adjustment for maternal smoking, abstinence and varicocele, sperm concentration and motility were significantly correlated to maternal serum BPA. No other associations of maternal serum BPA with another testicular function were observed. In another study aimed at evaluating the association between prenatal exposure to diethylhexyl phthalate (**DEHP**) / diisononyl phthalate (**DiNP**) and reproductive parameters of adolescent men, it was found that some metabolites of these phthalates were negatively associated with male reproductive functions such as testicular volume and reproductive hormone levels (FSH and LH) but not with semen quality [139]. Other male reproductive traits have been shown to be related to EDC exposure in fetal life and those include genital malformations such as cryptorchidism and hypospadias [140], testicular cancer [5] and anogenital distance [141]. In summary, there are a relatively small number of longitudinal studies assessing the association between prenatal exposure to EDCs and semen quality [142–144]. The correlation is still unclear except for a few rare cases of occupational or environmental accidents like in Seveso and Taiwan, where a clear significant association was observed between prenatal exposure and semen quality during adulthood. However, in both these studies, the number of participants was small and exposure levels in women living in the contaminated area overlapped with the background exposure [142].

#### Postnatal exposure to EDCs

The studies evaluating the association between EDC exposure during adulthood and semen quality are much more abundant because they are logistically and financially less challenging. An extensive review of the most relevant studies evaluating this relationship has recently been published, although it must be acknowledged that the results obtained for most of the evaluated EDCs are inconclusive due to the extreme heterogeneity of the reports [143]. In summary, contrary to previous evidence [145], recent studies seem to support the potential link between BPA exposure and low semen quality. A significant negative association between urinary BPA levels and sperm concentration as well as sperm count was observed in 215 Spanish university students [146] and a subgroup of obese Chinese men [147]. Data on PCBs and dioxins also confirm a negative relationship between exposure and semen quality. Higher quartiles of Russian men exposed to TCDD had lower sperm concentration, sperm count and sperm motility [148]. A study following the YuCheng accident in Taiwan found that similarly to the men that were prenatally exposed to PCBs, men who were exposed in adulthood also had a lower sperm morphology [149]. Another detailed review also concluded that exposure to PCBs and polychlorinated compounds during adult life seem to be negatively associated with sperm motility and sperm morphology, respectively [130]. Concerning the effects of phthalates, mixed results exist but most of them indicate a negative association between exposure and semen quality. Out of three recent cross-sectional studies, two demonstrated a negative association between urinary or seminal phthalate levels and semen quality [150, 151], whereas one did not [152]. Smarr et al. found an association between phthalates measured in seminal plasma of 339 men and decreased semen volume, sperm motility, viability and morphological aberrations [151]. Similarly, a significant adverse association was observed between 11 urinary phthalate metabolites levels and sperm concentration [150]. However, in the study of Albert et al., there was no association between urinary phthalate metabolite and sperm quality parameters [152]. The results on perfluorinated compounds such as PFAAs are still very contradictory since one study on Faroese men found no association between serum PFAA levels and semen variables [153], whereas another study on Chinese men found a negative association between levels of PFAA and sperm motility [154]. Results on polybrominated diphenyl ethers (**PBDE**) are also mixed and it is difficult to draw conclusions on their effects on semen quality [155, 156].

## Conclusion

Identifying the multiple causes behind the increasingly low semen quality is very challenging. Firstly, the field of male fertility is underfunded and has not been receiving great attention since the advancement reached in assisted reproductive technique. With the intracytoplasmic sperm injection (**ICSI**) only one sperm is sufficient to overcome male infertility. This has dramatically reduced intellectual interest in the underlying etiology of male infertility and the development of non-invasive therapeutic strategies that target the male patient [157]. Secondly, epidemiological evidence demonstrating a clear association between specific environmental and lifestyle factors is still limited (e.g. smoking, stress, EDCs, etc.) and their effects on spermatogenesis are generally more subtle than major. Exposure to these factors may occur alone or in combination during the fetal period, reflecting the maternal lifestyle, or during adulthood. Prenatal exposure may affect testis development and potentially exacerbate adverse effects on spermatogenesis related to adult exposure to other environmental and lifestyle factors. Accurately dissecting the impact of each factor is extremely complex because doses and periods of exposure vary as does the combination of factors to which each individual is exposed to. With the notable exception of prospective studies, it is also difficult to obtain prenatal exposure records and evaluate the presence of other confounding factors several decades before the reproductive defects are diagnosed. Finally, each individual is unique both in terms of genotype and environment. This means that any adverse effect of environmental or lifestyle factors on spermatogenesis will not have the same impact on individuals; it will nevertheless affect the study design, the interpretation of data and complicate the ability to provide epidemiological evidence. Understanding how environmental factors impinge on male reproductive health and spermatogenesis will continue to rely on the interpretation of epidemiological and animal studies. However, we are witnessing the emergence of new avenues to existing approaches that will improve our comprehension of the environmental exposure affecting male reproductive health. With regard to epidemiological analyses, we believe that the implementation of large-scale prospective cohort studies will be crucial to obtain accurate records of exposure and avoid the biases associated with traditional retrospective studies. Similarly recent developments in metabolomics and steroidomic could be used to boost our analytical power by identifying new set of biomarkers present in biological fluids associated with poor semen parameters. As far as experimental studies are concerned, the majority of them are based on rodent experiments despite the significant differences between humans and rodents in testicular development and reprotoxic effects. These inter-species differences forced the scientific community to develop more relevant in vitro approaches utilizing human tissues [158]. In the past few years, we have seen the emergence of new model systems such as in vitro or xenograft approaches using human fetal testis at human-relevant doses that can bridge the gap between direct evidence from animal experimental models and indirect evidence based on epidemiological data. Finally, one should not forget that male infertility is often multifactorial in origin and caused by both genetic and extrinsic factors. Although the focus of this review is on environmental factors, we still underestimate the genetic factors of male infertility responsible for morphological, qualitative or functional sperm defects [159]. So far relatively few genes have currently been identified, especially in severe cases of azoospermia, teratozoospermia. With the advent of whole exome sequencing (WES) and whole genome sequencing (WGS) applied to the study of large cohorts of cosanguinous patients with sperm abnormalities, it is highly likely that dozens of new genes or gene mutations affecting semen quality will be identified.

## Data Availability

Not applicable.

## Abbreviations

BPA: Bisphenol A
DDE: Diphenyl-dichloro-ethylene
DDT: Dichlorodiphenyltrichloroethane
DEHP: Diethylhexyl phthalate
DES: Diethylstilbestrol
DiNP: Diisononyl phthalate
EDCs: Endocrine disrupting Chemicals
EMF: Electro Magnetic Fields
FSH: Follicle stimulating hormone
ICSI: intracytoplasmic sperm injection
LH: Luteinizing hormone
Mio: Million
PBB: Polybrominated biphenyls
PBDE: Polybrominated diphenyl ethers
PCBs: Polychlorinated Biphenyls
PCDF: Polychlorinated dibenzofurans
PCFs: Perfluorinated compounds
PFOA: Perfluorooctanoic acid
POPs: Persistent Organic Pollutants
PFAAs: Perfluorinated alkyl acids
RF-EMF: Radio Frequency - Electromagnetic Fields
ROS: Reactive Oxygen Species
SCO: Sertoli-Cell Only
SLE: Stressful Life Events
TCDD: 2,3,7,8-tetrachlorodibenzo- p-dioxin
TDS: Testicular Dysgenesis Syndrome
WHO: World Health Organization
